# PHF11 expression and cellular distribution is regulated by the Toll-Like Receptor 3 Ligand Polyinosinic:Polycytidylic Acid in HaCaT keratinocytes

**DOI:** 10.1186/s12865-015-0131-y

**Published:** 2015-11-14

**Authors:** Pauline Muscat, Karessa Mercado, Kathryn Payne, Hardip Chahal, Graham Jones

**Affiliations:** School of Science and Health, Western Sydney University, Locked Bag 1797, 2751 Penrith, NSW Australia; Present address: Garvan Institute for Medical Research, Darlinghurst, NSW Australia

**Keywords:** PHF11, keratinocyte, HaCaT, poly(I:C), TLR3, claudin-1

## Abstract

**Background:**

Inflammatory skin diseases such as atopic dermatitis and psoriasis represent a complex interaction between the skin and infiltrating immune cells, resulting in damage to the skin barrier and increased inflammation. Polymorphisms in *PHF11* have been associated with dermatitis and allergy and PHF11 regulates the transcription of T-cell cytokines as well as class switching to IgE in activated B-cells. The importance of skin barrier homeostasis in the context of inflammatory skin diseases, together with reports identifying *PHF11* as an interferon-induced gene, have led us to examine its role in the innate immune response of keratinocytes.

**Results:**

We developed a cell culture model that allowed us to analyze the effects of the double-stranded RNA analogue poly(I:C) on a confluent cell monolayer immediately after a 24-h treatment, as well as three days after withdrawal of treatment. Immediately after treatment with poly(I:C), *PHF11*, *IL8,* and interferon-dependent *ISG15* RNA expression was increased. This was accompanied by nuclear localization of PHF11 as well the tight junction protein claudin-1. Knock-down of PHF11 resulted in increased interleukin-8 expression and secretion immediately following treatment with poly(I:C), as well as changes in the cellular distribution of membrane-bound and increased nuclear claudin-1 that was observed up to 3 days after the withdrawal of poly(I:C). This was associated with lower cell density and a decrease in the number of cells in the G1 phase of the cell cycle.

**Conclusions:**

In addition to a role for PHF11 in lymphocyte gene expression, we have now shown that PHF11 was part of the keratinocyte innate immune response by poly(I:C). As knock-down of PHF11 was associated with increased expression of the pro-inflammatory chemokine IL-8 and changes in the cellular distribution of claudin-1, a change normally associated with increased proliferation and migration, we suggest that PHF11 may contribute to epidermal recovery following infection or other damage.

## Background

Plant homoeodomain finger protein 11 (PHF11) is highly expressed in circulating immune cells, with increased expression in T-helper 1 (Th1) T-cells relative to Th2 T-cells [1]. Knock-down of PHF11 using small interfering RNA (siRNA) decreases expression of the interferon-γ (*IFNG*) gene in Th1 cells [1] through a mechanism that involves a reduction in NFκB-dependent transcriptional activity [1, 2]. A role for PHF11 in *IFNG* gene expression, and more recently the finding that PHF11 increases class switch recombination to IgE in murine B-cells [3], supports a role for PHF11 in allergic disease. A link between *PHF11* and allergic disease was shown in earlier genetic linkage and association studies [4–6], with alternate alleles of a single nucleotide polymorphism in the 3’ non-translated exon of *PHF11* associated with a change in the expression of this gene in Th1 cells [1] through differential binding of the transcription factor Oct-1 [7]. Although recent genome-wide association studies (GWAS) of asthma and atopic dermatitis have not supported a genetic association between *PHF11* and allergy, it remains possible that there may be an association in selected cohorts of severely affected individuals who show a very early age of onset with highly elevated IgE levels and who are more likely to require treatment by specialist clinicians [8].

Allergic asthma and dermatitis are characterized by immune sensitization, which refers to the initial recognition of an antigen by the immune system and the production of antigen-specific IgE antibodies. In susceptible individuals any subsequent exposure to the same allergen will result in a robust and aggressive immune response. It is now apparent that there is a link between immune sensitization and the integrity of the skin barrier. As an example, filaggrin is a protein found in the outermost layer of the epidermis called the stratum corneum and is essential for the integrity of the skin barrier (for review, see [9]). Mutations in the gene encoding filaggrin (*FLG*) are associated with immune sensitization in allergic dermatitis and asthma [10, 11] and these mutations cause an impairment of the skin barrier function [12, 13]. Genetic association between a locus containing the gene *C11orf30* is highly reproduced in GWAS of asthma and dermatitis [14–16], and although the functional relationship between *C11orf30* or other nearby genes with asthma and dermatitis is not understood, the protein product of *C11orf30* (EMSY) is important in epithelial tumours of the breast and ovary [17, 18].

In addition to a genetic basis for a compromised skin barrier, elevated expression of Th2-type cytokines such as interleukin (IL)-4 and IL-13 in the skin of individuals with atopic dermatitis decreases filaggrin expression [19]. These cytokines also decrease the expression of the tight junction protein claudin-1 in the skin of individuals with atopic dermatitis [20]. The combination of genetic and inflammatory triggers that result in a decrease in the integrity of the skin barrier are also linked to the high rate of bacterial and viral skin infections of individuals with atopic dermatitis [21, 22]. Keratinocytes express several members of the Toll-like Receptor (TLR) family that are pattern recognition receptors for viral and bacterial pathogens. The TLR3 recognizes double-stranded RNA that is a replication intermediate for a number of viruses, as well as RNA that is released from damaged cells. Activation of TLR3 is an important part of the keratinocyte innate immune response [23], as well as the repair and maintenance of the skin barrier [24, 25].

A review of the literature revealed that *PHF11* is an interferon stimulated gene (ISG) and that its expression is increased following infection by several different viruses [26–29]. Although previous functional studies of *PHF11* have centered on its role in the regulation of cytokine gene expression in T-lymphocytes [1, 2], the susceptibility of individuals with atopic dermatitis to viral infection and the finding that *PHF11* is an ISG has led us to test for *PHF11* expression in keratinocytes and whether its expression is regulated by polyinosinic:polycytidylic acid (poly(I:C)), a ligand for TLR3 and an analogue of double-stranded RNA.

In this study we report that *PHF11* is expressed in the human HaCaT keratinocyte cell line and that treatment of this cell line with the double-stranded RNA (dsRNA) analogue poly(I:C) resulted in an increase in *PHF11* expression and the localization to the nucleus of the PHF11 protein. Furthermore, siRNA knock-down of *PHF11* mRNA resulted in a loss of PHF11 from the nucleus and this was accompanied by an increase in IL-8 expression, altered appearance of claudin-1 at the cell membrane and the appearance of claudin-1 in the nucleus. We suggest that in addition to its role in circulating T-cells, PHF11 also plays a role in the innate immune response of keratinocytes and that a reduction in *PHF11* expression may contribute to inflammation and tissue remodeling following infection or tissue damage.

## Methods

### Cell culture

HaCaT Keratinocytes were grown in a complete cell culture medium consisting of Dulbecco’s Modified Eagle Medium (DMEM) and 10 % fetal bovine serum (FBS) at 37 °C in a humidified atmosphere containing 5 % CO_2_.

### cDNA synthesis and quantitative real-time PCR

Total RNA was harvested using the PureLink® RNA Mini kit (Ambion®/Life TechnologiesTM, Austin, TX, USA) and cDNA was synthesized from 1 μg of RNA using the Applied Biosystems High-Capacity cDNA Reverse Transcription Kit according to the manufacturers instructions. Quantitative real-time PCR was done using SYBR® PCR Master Mix (Applied Biosystems, Austin, TX, USA), 0.1 μM of forward and reverse primers in a final volume 10 μl. Reactions were transferred to an Illumina Eco 48-well plate and analysed using an Illumina® Eco real-time PCR system. Primer sequences are shown in Table 1.

**Table 1:** Primers used for Quantitative real-time PCR

Primer	Product size (bp)	Sequence (5’-3’)
		Forward (F), Reverse (R)
Claudin-1	109	F: GGTCAGGCTCTCTTCACTGG
		R: GCCTTGGTGTTGGGTAAGAG
IL-8	109	F: TCTGCAGCTCTGTGTGAAGG
		R: AAATTTGGGGTGGAAAGGTT
ISG15	97	F: AGCATCTTCACCGTCAGGTC
		R: GAGAGGCAGCGAACTCATCT
KRT10	91	F: CCTGGCTTCCTACTTGGACA
		R; TTGCCATGCTTTTCATACCA
KRT14	90	F: TCCTCAGGTCCTCAATGGTC
		R: CGACCTGGAAGTGAAGATCC
KRT1	110	F: CAACCAGAGCCTTCTTCAGC
		R: AGGAGGCAAATTGGTTGTTG
PHF11	140	R: TCCTGCTTCCTTGCATTTCT
		F: GGAAGGAAGAAACCCCTCTC
SDHA	86	F: TGGGAACAAGAGGGCATCTG
		R: CCACCACTGCATCAAATTCATG

### siRNA knockdown

All siRNAs have been previously tested and validated [2]. The sequence of PHF11-specific siRNAs are: siRNA siRNA_1 CACCGTGGGATGTGATTTAAA (Qiagen, Hilden, Germany, cat no. SI00113554) and siRNA_5 ATCATCGCTCAAAGTGCTAAA (Qiagen, cat no. SI03047198), mapping to exons 4 and 5 of PHF11 isoform NM_001040443.1, respectively. On the day of transfection, HaCaT keratinocytes were harvested and resuspended at a concentration of 1 × 10^5^ cells in 300 μl of complete cell culture media. 600 ng of siRNA was diluted in 100 μl of Optimem culture media (Gibco®/Life Technologies, Austin, TX, USA), followed by the addition of 6 μl of Hiperfect® Transfection reagent (Qiagen, Hilden, Germany). Following a 10 min incubation at room temperature, the siRNA mixture was combined with the HaCaT cells and plated directly onto 24-well plates (1 × 10^5^ cells/well) or an 8-well Nunc® Lab-Tek® Chamber slide (5 × 10^4^ cells/well) and incubated overnight at 37 °C/5%CO_2_. The transfection solution was then replaced with complete cell culture medium and the cells were returned to the incubator for a further 2 days, and then treated with poly(I:C) for 24 h. Cells were harvested either 24 or 96 h after treatment with poly(I:C).

### HEK Cell transfection and PHF11 expression plasmids

Full-length PHF11 cDNA was cloned into the plasmid expression vector pEGFP-C1 (Clontech) and was then used as a template to generate the two C-terminal deletion constructs del218 and del165 that terminate at valine residue 218 and alanine residue 165, respectively. Numbering of amino acids is based on NCBI reference sequence NP_001035533.1. Transfection of HEK cells was done as previously described [1]. Identification of a putative nuclear localization sequence was done using the following online tool: http://nls-mapper.iab.keio.ac.jp/cgi-bin/NLS_Mapper_form.cgi. Western blot analysis confirmed that each recombinant protein was expressed at the predicted molecular weight (data not shown). Images were analysed using ImageJ (http://imagej.nih.gov/ij/).

### Immunofluorescence

In experiments to visualize IL-8 production, 0.5 mM Monensin sodium salt (Sigma, St. Louis, USA) was added to the culture media and cells returned to 37 °C/5%CO_2_ for the final 4 h of stimulation by poly(I:C) [30]. To visualize IL-8, as well as claudin-1 and PHF11, cells were fixed in a 4 % formaldehyde solution for 10 min at room temperature, washed and permeabilized by washing for 3 × 5 min in PBS/0.01 % Triton X100. Cells were then blocked in Image-iT™ FX Signal Enhancer (Molecular Probes, Oregon, USA) for 30 min and washed a further 3 × 5 min in PBS/0.01 % Triton X100. The cells were incubated with the following primary antibodies in blocking buffer (1%BSA, 0.01 % Triton X100, 5 % FCS in PBS) overnight at 4 °C: rabbit anti-Claudin 1 (1:1000, Sigma-Aldrich, SAB4503546), rabbit anti-PHF11 (1:100, ProteinTech Group, 10898-1-AP), mouse anti-CXCL8/IL-8 (10 μg/ml, R&D systems, MAB208). Cells were then washed for 3 × 5 min in PBS/0.01 % Triton X100 and then incubated with a 1:1000 dilution Alexa Flour® 488 goat anti-rabbit IgG (H + L) and/or a 1:1000 dilution of Alexa Flour® 555 goat anti-mouse IgG (H + L) (Molecular probes/Life Technologies™, Austin, TX, USA) in blocking buffer at room temperature for 1 h in the dark. Cells were washed 2 × 5 min in PBS/0.01 % Triton X100, and nuclear DNA was stained using a solution of 1 μg/ml Hoechst 33342 (Molecular probes/Life Technologies™, Austin, TX, USA) for 10 min at room temperature protected from light. After a final 5 min wash in PBS/0.01 % Triton X100, 2 drops of ProLong® Gold antifade reagent was added to the slide and covered with a glass cover slip. Slides were viewed on an Olympus BX43 Microscope fitted with an X-Cite Series 120Q EXFO Halogen Lamp using cellSens standard software or using a TCS-SP5 confocal microscope (Leica Microsystems, Germany).

### IL-8 enzyme-linked immunosorbent assay (ELISA)

Cells were transfected with siRNA as described and 1 × 10^4^ cells were plated per well in a 96-well plate in triplicate. Following 24 h treatment with poly(I:C), the concentration of secreted IL-8 was determined using the Human CXCL8/IL-8 Quantikine ELISA (R & D Systems, MN, USA, Cat no. D8000C). The amount of secreted IL-8 was normalized to cell number using the CyQUANT NF Cell Proliferation Assay Kit (Molecular probes/Life Technologies™, Austin, TX, USA). Results represent the outcome of 4 independent siRNA transfections.

### Cell cycle analysis

At the conclusion of an experiment, cell culture media was removed and the cells harvested using trypsin. Cells were washed once in 5 ml of PBS, and then resuspended and fixed in 5 ml of 70 % (v/v) ethanol (Sigma-Aldrich) at 4 °C for up to 7 days. Following fixation, cells were centrifuged at 300 × g for 5 mins at room temperature, washed in PBS, and then resuspended in a solution of 1 μl RNase, 2 μl 10x propidium iodine (PI) (Sigma-Aldrich) in PBS and incubated at 37 °C for 50 min. Cells were then collected by centrifugation at 300 × g for 5 min at room temperature and the cell pellet resuspended in 1 ml of PBS. Samples were analysed using a FACSCanto II (BD Bioscience, Franklin Lake NJ, USA). Ten thousand events were collected per sample and data was exported and analysed using FlowJo Flow Cytometry Analysis Software 7.6.5 (Tree Star, Ashland, OR, USA). A scatterplot was created and the region containing the G1, S and G2/M peaks was gated to select singlet cells. A histogram of the gated region was produced and live cells as well as sub-G1 cells were defined. Cell cycle analysis was done using the Dean-Jett-Fox model.

## Results

### Poly(I:C) increased the expression and nuclear localization of PHF11

The HaCaT cell line was used to test whether poly(I:C) regulates the expression of *PHF11* in keratinocytes. The induction of interferon signaling by poly(I:C)-dependent activation of toll-like receptor 3 (TLR3) has previously been reported in this cell line [23, 31]. Since poly(I:C) is able to activate innate immune signaling pathways as well as induce apoptosis [32], we were interested in examining the regulation of *PHF11* to poly(I:C) at 1 and 3 days after the commencement of treatment.

**Fig. 1:**
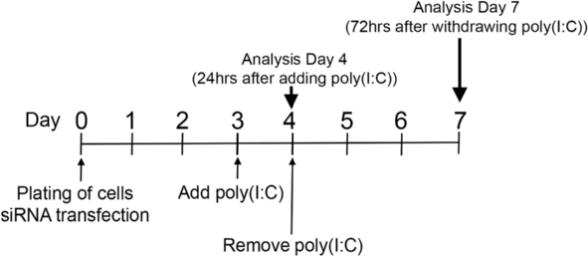
Summary of siRNA transfection and culturing of HaCaT keratinocytes. Cells were cultured for up to 7 days, with poly(I:C) treatment commencing on day 3 of culture for 24 h. Cells were analyzed immediately after treatment, or were washed and cultured for a further 3 days. Transfection with siRNA was done on the day of plating

To do this, HaCaT keratinocytes were cultured for 3 days to reach a confluent monolayer and then treated for 24-h with poly(I:C). Cells were either analyzed immediately (i.e., 24 h after commencing treatment; a total of 4 days in culture) or poly(I:C) was removed and cells cultured for a further 3 days, making a total of 7 days in culture (Fig. 1).

**Fig. 2:**
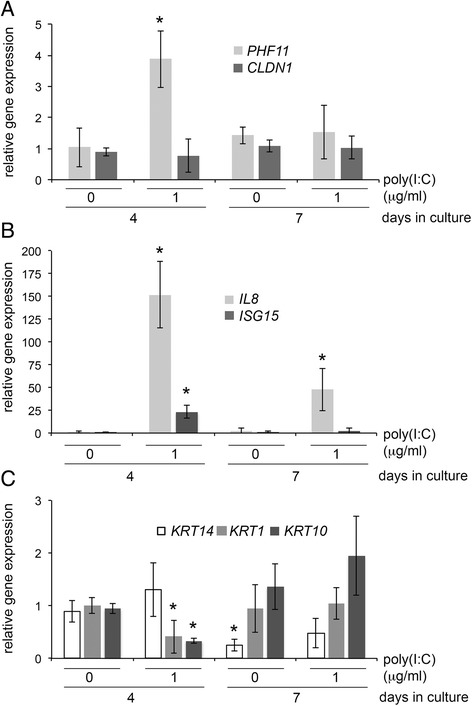
Profile of gene expression in HaCaT keratinocytes in response to poly(I:C) at 4 and 7 days in culture. Cells were left untreated or treated with 1 μg/ml of poly(I:C) on day 3 and harvested on day 4 or on day 7. Quantitative real-time PCR was used to analyse the expression of PHF11, claudin-1 (*CLDN1*), IL-8 (*IL8*), interferon-stimulated gene 15 (*ISG15*) and keratins 1, 14 or 10 (*KRT1*, 10, 14). Results represent 4 independent experiments. Bars indicate average ± standard deviation, asterisks indicate significant difference relative to control (*p* < 0.05, Mann–Whitney *U*-test)

Analysis of gene expression 24 h after commencing treatment with 1 μg/ml of poly(I:C) showed an approximately 4-fold increase in *PHF11* RNA (Fig. 2a, 1 μg/ml poly(I:C), 4 days in culture) relative to untreated cultures. Three days after withdrawal of poly(I:C), expression of *PHF11* had declined to be no different from cultures not treated with poly(I:C) (Fig. 2a, 1 μg/ml poly(I:C), 7 days in culture). There was no change in the expression of RNA encoding the tight junction marker claudin-1 (*CLDN1*) between different treatment conditions (Fig. 2a, CLDN1). To confirm that the cells had responded to poly(I:C), we also analyzed the expression of genes encoding interleukin (*IL*)*-8* and *ISG15*, both of which are regulated by poly(I:C) [33, 34]. Analysis of RNA expression immediately after 24 h of treatment with poly(I:C) showed *IL8* was increased by more than 140-fold while *ISG15* expression was increased approximately 20-fold (Fig. 2b, 1 μg/ml poly(I:C), 4 days in culture). Analysis of RNA expression three days after withdrawal of poly(I:C) showed *ISG15* expression had declined to levels not different from untreated cultures, while IL8 RNA remained approximately 50-fold higher than that seen in untreated controls (Fig. 2b, *IL8*, 7 days in culture).

As the activation of TLR3 can promote skin barrier repair [25], we also looked for changes in the expression of keratin genes that are normally active in undifferentiated keratinocytes (keratin 14) or in keratinocytes during early differentiation (keratins 1 and 10). After 3 days in culture, treatment for 24 h with poly(I:C) did not change the expression of keratin-14, although the expression of both keratin-1 and −10 were decreased 2.5 to 3-fold under the same conditions (Fig. 2c, 4 days in culture). Assaying the expression of each keratin gene three days after the withdrawal of poly(I:C) revealed a significant decrease in the expression of keratin-14, independently of poly(I:C) treatment (Fig. 2c, 7 days in culture), whereas the expression of keratin-1 and keratin-10 were either not different from untreated controls or showed only a small increase in expression, respectively (Fig. 2c).

**Fig. 3:**
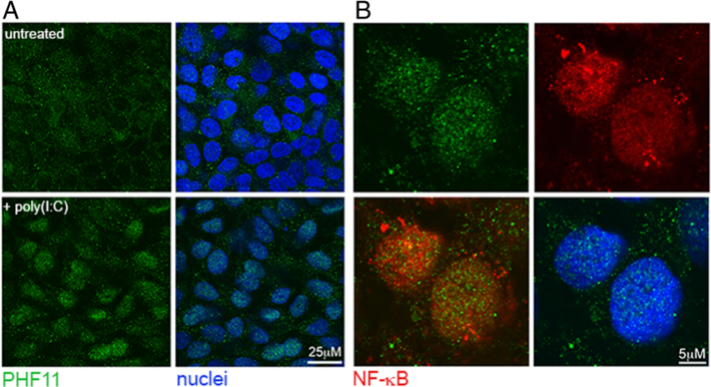
Nuclear localization of PHF11 is induced by poly(I:C). **a** Confocal microscopy of PHF11 localization in HaCaT keratinocytes on day 4 of culture in the absence of poly(I:C) (untreated), or after a 24-h poly(I:C) treatment (+poly(I:C)). **b** Higher magnification image of the nuclei of cells treated with poly(I:C) and stained for PHF11 (green), NF-κB (red). A merged image of PHF11 and NF-κB (lower left), as well as DAPI-stained nuclei (lower right) is shown

We have previously shown that in Jurkat T-cells PHF11 localization is mainly cytoplasmic, although stimulation with PMA/ionomycin increases the amount of PHF11 in the nucleus [2]. In HaCaT keratinocytes, nuclear localization of PHF11 was strongly increased following a 24 h incubation with poly(I:C) (Fig. 3a). Three days after withdrawal of poly(I:C) the nuclear localization of PHF11 had decreased and, similar to untreated cultures, showed strong cytoplasmic localization (data not shown). The distribution of PHF11 in the nucleus had a highly speckled or punctate appearance and differed from nuclear distribution of NF-κB (Fig. 3b).

**Fig. 4:**
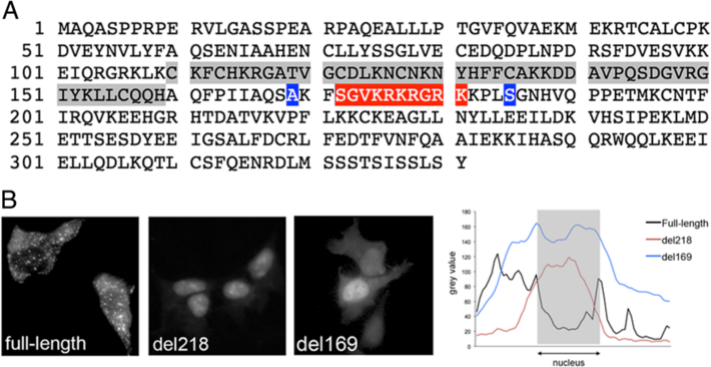
C-terminal deletion mutants of PHF11 are localized in the nucleus. **a** The amino acid sequence of PHF11 showing the PHD domain (grey highlight), predicted nuclear localization sequence (red highlight) and the location of the C-terminal deletion mutants del218 (S) and del169 (A) (blue shading). **b** *Left:* Cellular localization of full-length, del218 and del169 GFP-tagged PHF11 proteins in HEK cells. *Right:* plot of pixel density across cells expressing full-length or deletion mutants of PHF11. The position of the nucleus is indicated and the vertical axis indicates the pixel density (arbitrary units)

The stimulus-dependent nuclear localization of PHF11 led us to search for sequences that control its cellular localization. To do this, we first searched for possible nuclear localization sequences (NLS) using an online mapping tool (cNLS Mapper) [35]. This identified a putative monopartite NLS beginning at residue 172 and consisting of the sequence SGVKRKRGRK (Fig. 4a). Next, full-length PHF11 as well as two C-terminal deletion mutants that terminate at amino acids 218 and 169 (Fig. 4a) were cloned into a green fluorescent protein (GFP) expression vector. Transfection of human embryonic kidney (HEK) cells showed that the expressed full-length protein was present in the cytoplasm, as well as in a speckled pattern in the nucleus (Fig. 4b, full-length). Deletion to amino acid 218, which retained both the PHD domain as well as the putative NLS, resulted in nuclear localization (Fig. 4b, del218). However, the PHF11 deletion mutant that terminated at residue 169 and lacked the putative NLS also showed strong nuclear localization (Fig. 4b, del169). These results showed that the deletion of sequences in the C-terminal half of PHF11 between amino acids 218 and 331, but not of a sequence rich in basic residues located between residues 172 and 181, resulted in nuclear localization of PHF11.

### siRNA knock-down of PHF11: analysis immediately after poly(I:C) treatment

**Fig. 5:**
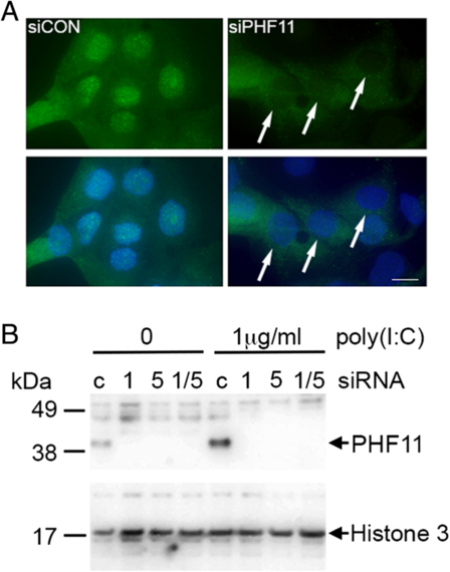
siRNA knock-down of PHF11 decreases nuclear PHF11 in poly(I:C) treated cells. Cells were transfected with control (siCon) or PHF11-specific (siPHF11) siRNA (day 1) and then treated with 1 μg/ml of poly(I:C) (day 3) for 24 h. **a**. Image of transfected cells showing an absence of PHF11 in cell nuclei. Arrows indicate the location of nuclei with reduced PHF11. Green: PHF11; blue: nuclei. Scale bar 10 μM. **b**. Western blot showing knock-down of PHF11 in nuclear lysates in the absence and presence of PHF11. Shown are the results for control (c) and two different PHF11-specific siRNAs either singly (1 & 5) or in combination (1/5)

To explore the role of PHF11 in keratinocytes, HaCaT cells were treated with siRNA that has previously been used to successfully knock-down *PHF11* expression in T-cells [2]. In HaCaT cells transfected with a control siRNA and treated with poly(I:C), PHF11 immunoreactivity was clearly visible in nuclei after 24 h of treatment with poly(I:C) (Fig. 5, siCon). Under the same conditions, transfection of HaCaT cells with *PHF11*-specific siRNA resulted in a loss of nuclear PHF11 (Fig. 5a). Western blot analysis detected an approximately 40 kDa PHF11-specific band in control siRNA transfected nuclei that increased following treatment with poly(I:C) (Fig. 5b, siRNA c) Two *PHF11*-specific siRNAs (1 & 5, Fig. 5b) were equally efficient at knocking-down PHF11 when transfected singly or in combination (Fig. 5b, 1/5). All siRNA experiments described in this paper use the combination of *PHF11*-specific siRNA 1 and 5.

**Fig. 6:**
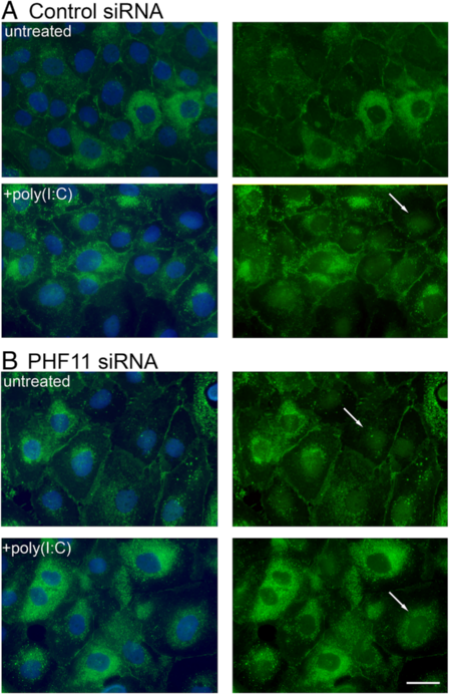
Knock-down of PHF11 increases nuclear claudin-1. Cells were transfected with either: **a**. control (control siRNA) or **b**. a mixture of PHF11 siRNA_1 & 5 (PHF11 siRNA) and left untreated or treated on day 3 with 1 mg/ml poly(I:C) for 24 h and analyzed immediately thereafter. Shown are representative images of claudin-1 and nuclei (left hand column) or claudin-1 only (right hand column) from one of three independent experiments. Green: claudin-1; blue: nuclei. Arrows indicate nuclear claudin-1. Scale bar 20 mM

We next examined the expression and localization of the tight junction marker claudin-1 in siRNA-transfected cells. In cells transfected with control siRNA and cultured for a total of 4 days but not treated with poly(I:C), claudin-1 immuno-reactivity was visible at the membrane with some cells displaying intense intracellular claudin-1 immuno-reactivity (Fig. 6a, untreated). Treatment with poly(I:C) resulted in increased intracellular claudin-1, as well as the appearance of claudin-1 in the nucleus (Fig. 6a, +poly(I:C)). In parallel cultures transfected with *PHF11*-specific siRNA but not treated with poly(I:C), there was an increase in intracellular claudin-1 as well as evidence of nuclear claudin-1 (Fig. 6b, untreated). Treatment of cells transfected with *PHF11*-specific siRNA with poly(I:C) reduced membrane claudin-1 and increased the number of cells displaying intracellular claudin-1 immuno-reactivity (Fig. 6b, +poly(I:C)).

**Fig. 7:**
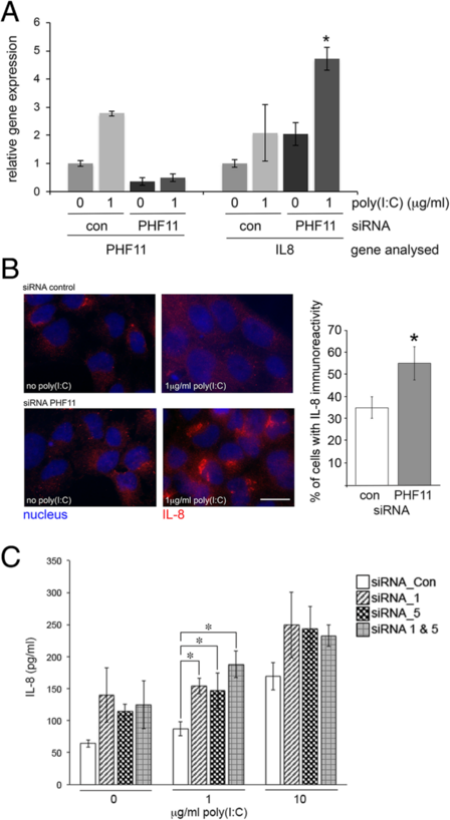
Knock-down of *PHF11* expression increases IL-8 expression immediately after treatment with poly(I:C). **a** Quantitative real-time PCR analysis of *IL8* RNA in cells transfected with control (con) or PHF11-specific (PHF11) siRNA. Results are normalized to untreated cells transfected with control siRNA. **b** Immunohistochemistry of IL-8 synthesis. Red: IL-8; Blue: nuclei. Quantification of IL-8 positive nuclei is shown on the right. Asterisks indicate significant difference relative to control (*p* < 0.05, Mann–Whitney *U*-test). **c** ELISA quantification of IL-8 secretion from siRNA-transfected HaCaT cells. Cells were transfected with control (siRNA_Con) or *PHF11*-specific siRNA (siRNA_1, _5, and a combination of 1 & 5) and then treated with poly(IC) (1 or 10 μg/ml) for 24 h prior to assaying for secreted IL-8. Results represent 4 independent experiments; asterisks indicate significant differences (*p* < 0.05, Kruskal-Wallis test). Scale bar 20 μM

Assaying poly(I:C)-dependent gene expression showed there was no difference in the expression of *ISG15* between cells transfected with either control or *PHF11*-specific siRNA and this was independent of treatment with poly(I:C) (data not shown). However, knock-down of *PHF11* expression resulted in an approximately 2-fold greater increase in poly(I:C)-dependent *IL8* expression 24 h after adding poly(I:C) (Fig. 7a, 4.7 ± 0.2 Vs 2.1 ± 1.2, respectively), relative to cells transfected with control siRNA. The expression of IL-8 in cells transfected with control or *PHF11*-specific siRNA was next assayed by immuno-fluorescent imaging of monensin-treated cells and by quantitative ELISA. Analysis of fixed, monensin-treated cells revealed a significant increase in IL8 immunoreactivity in cells transfected with *PHF11*-specific siRNA (Fig. 7b). Quantitative ELISA analysis of IL-8 secretion from siRNA-transfected HaCaT cells showed knock-down of PHF11 resulted in a significant 1.8 to 2.1-fold increase in IL-8 secretion at 1 μg/ml of poly(I:C) (Fig. 7c). Knock-down of PHF11 also resulted in a non-significant 1.5-fold increase in secretion of IL-8 in HaCaT cells treated with 10 μg/ml of poly(I:C), relative to control siRNA (Fig. 7c).

### siRNA knock-down of PHF11: analysis 3 days after poly(I:C) treatment

**Fig. 8:**
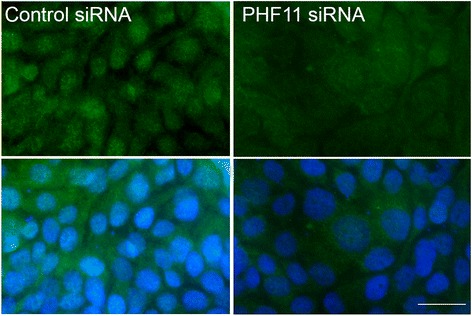
PHF11 expression in siRNA-transfected cells on day 7 of culture, 3 days after withdrawal of poly(I:C). *Left panels:* cells transfected with control siRNA; *Right panels:* cells transfected with PHF11-specific siRNA. Green: PHF11; Blue: nuclei. Scale bar 20 μM

We first ascertained whether any differences could be detected in PHF11 expression between cells transfected with control and *PHF11*-specific siRNA at day 7 in culture (3 days after withdrawal of poly(I:C)). In cells that had been transfected with control siRNA, nuclear accumulation of PHF11 was still evident against a background of PHF11 expression (Fig. 8, *Left hand panels*). In contrast, cells that had been transfected with *PHF11*-specific siRNA show a uniform level of PHF11 expression with no evidence of concentration in the nucleus (Fig. 8, *Right hand panels*).

**Fig. 9:**
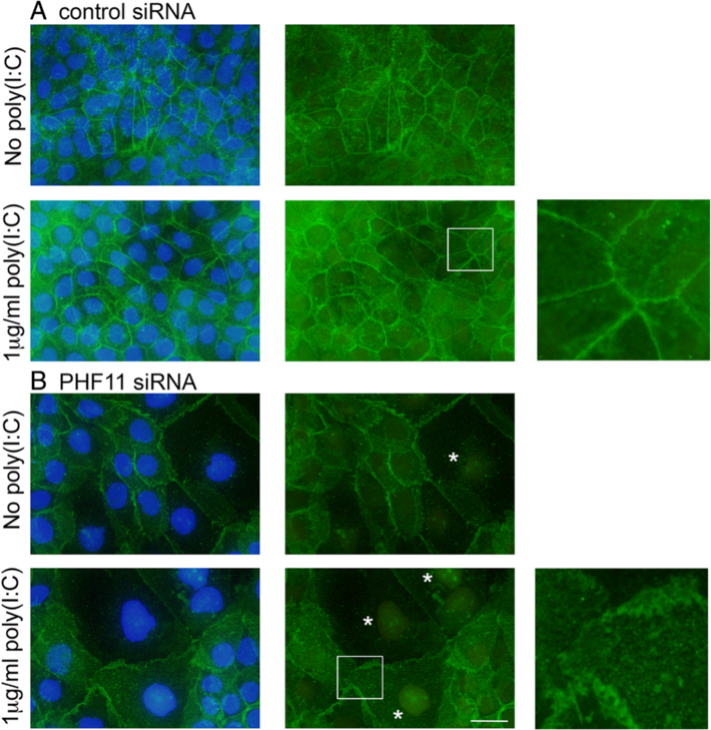
Distribution of claudin-1 in siRNA transfected cells after 7 days in culture. Cells were transfected with control or PHF11-specific siRNA as already described, treated with poly(I:C) and analyzed 3 days thereafter (a total of 7 days in culture; see Fig. 1). Enlarged images of the boxed areas are shown on the right of the figure and nuclei showing claudin-1 immuno-reactivity are indicated by asterisks. Shown are representative images of three independent experiments. Green: claudin-1; blue: nuclei. Scale bar 20 μM

After 7 days in culture, claudin-1 immunoreactivity was clearly visible at the cell membrane of cells transfected with control siRNA with this distribution independent of prior treatment with poly(I:C) (Fig. 9a, control siRNA). Knock-down of *PHF11* RNA resulted in decreased cell density and increased cell size while claudin-1 immuno-reactivity at the membrane exhibited a more irregular appearance when compared to cells transfected with control siRNA (Fig. 9a & b, boxed and enlarged areas). Both effects were independent of prior treatment with poly(I:C) (Fig. 9b). Nuclear claudin-1 immunoreactivity was also observed in cells transfected with *PHF11*-specific siRNA with nuclear claudin-1 was observed more frequently in cells treated with poly(I:C) (Fig. 9b, PHF11 siRNA, asterisks). No difference in the expression of IL-8 was observed between control and PHF11-specific siRNA 3 days after the withdrawal of poly(I:C) (data not shown).

**Fig. 10:**
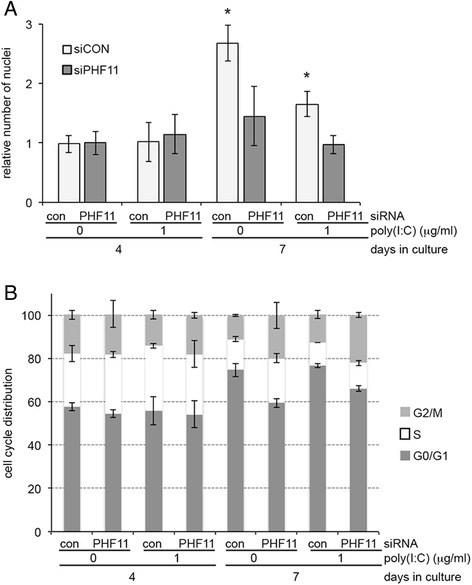
Knock-down of *PHF11* expression decreases cell number and the proportion of cells in the G1 phase of the cell cycle on day 7 of culture. **a** Cell number normalized to untreated cultures on day 4. **b** Cell cycle analysis of siRNA transfected cells in the treated or untreated with poly(I:C) on day 4 or 7 of culture. Con: control siRNA; PHF11: *PHF11*-specific siRNA. Results are from 3 independent experiments and show average ± standard deviation, asterisks indicate significant difference relative to control (*p* < 0.05, Mann–Whitney *U*-test)

An analysis of cell number in cultures transfected with control or *PHF11*-specific siRNA, and treated or not treated with poly(I:C), showed no difference in cell numbers on day 4 of culture (Fig. 10a, 4 days in culture). However, relative to day 4, on day 7 of culture there was a greater than 2.5-fold increase in cell number for cultures transfected with control siRNA but not treated with poly(I:C), with a smaller 1.7-fold increase in cell number when treated with poly(I:C) (Fig. 10a, 4 & 7 days in culture, con siRNA). In contrast, for cultures transfected with *PHF11*-specific siRNA there was no significant increase in cell number, irrespective of treatment with poly(I:C) (Fig. 10a, 4 & 7 days in culture, PHF11 siRNA). To investigate this further, we next analyzed the cell cycle distribution of cells transfected with control or *PHF11*-specific siRNA.

After 4 days in culture there was no difference in the cell cycle distribution of cells transfected with either control or *PHF11*-specific siRNA or between cultures treated or not treated with poly(I:C) (Fig. 10b, 4 days in culture). After 7 days in culture there was an increase in the percent of cells in G1 phase in cells transfected with control siRNA that was independent of poly(I:C) treatment (Fig. 10b, siRNA con, 4 Vs 7 days in culture: no poly(I:C), 57.8 ± 1.9 Vs 74.7 ± 3; 1 μg/ml poly(I:C), 55.8 ± 1 Vs 76.5 ± 1). In contrast, transfection with *PHF11*-specific siRNA resulted in significantly fewer cells in G1 after 7 days in culture in culture not treated or treated with poly(I:C) (Fig. 10b, siRNA PHF11, 7 days in culture: no poly(I:C), 59.4 ± 2; 1 μg/ml poly(I:C), 66.1 ± 1.3).

## Discussion

Extending on our previous work showing increased nuclear localization of PHF11 in activated T-cells [2], we show here that stimulation of HaCaT keratinocytes by poly(I:C) increased *PHF11* RNA as well as the nuclear localization of PHF11. The distribution of PHF11 between the cytoplasm and the nucleus was dependent upon a region that included a putative NLS that was distinct from the single PHD finger. Knock-down of *PHF11* led to an increase in IL-8 expression immediately following poly(I:C) treatment. A decrease in cell number, redistribution of claudin-1 within the cell membrane and an increased frequency of claudin-1 in the nucleus was seen three days after the withdrawal of poly(I:C).

In the HaCaT cell line, poly(I:C) induces apoptosis in a caspase-8 dependent manner [32], as well as inducing the transcription and synthesis of IL-8 [23]. Normal keratinocytes and the HaCaT cell line synthesize IL-8 and express the IL-8 receptors CXCR1 and CXCR2 [36, 37], allowing IL-8 to act as an autocrine factor for keratinocyte migration and proliferation [36], in addition to promoting the recruitment of neutrophils to a wound site [38]. The binding of dsRNA to TLR3 on keratinocytes initiates signaling pathways that include the activation of the NF-κB transcription factor as well as anti-viral interferon-dependent pathways. The cellular response to intracellular influenza A virus and extracellular poly(I:C) is very similar in lung epithelial cells, although adding poly(I:C) directly to the cell culture media, as was done in the study reported here, may also mimic the release endogenous cellular or viral dsRNA from damaged cells and the activation of TLR3 [39].

Consistent with a pro-inflammatory and pro-apoptotic role for poly(I:C) on cultured keratinocytes, poly(I:C) treatment leads to the loss of tight junctions from airway epithelial cells [40]. However, poly(I:C) also increases the expression of genes involved in skin barrier formation in cultured keratinocytes [25], while topical application of poly(I:C) accelerates wound healing in mice through the production of CXCL2 and the recruitment of neutrophils and macrophages to the wounded site [41]. Given the range of these effects and their importance to epithelial damage and repair, it is important to identify genes involved in TLR3-dependent signaling pathways that mediate the response to poly(I:C).

In experiments described here, HaCaT cells were transfected with siRNA and then two days later treated with poly(I:C) for 24 h, with knock-down of *PHF11* expression correlated with an increase in poly(I:C)-dependent IL-8 expression. Three days after withdrawal of poly(I:C) we observed marked differences in the appearance of cultures transfected with control or *PHF11*-specific siRNA. The distribution of membrane-localized claudin-1 in cells transfected with *PHF11*-specific siRNA was discontinuous and irregular and was highly reminiscent of the “zigzag” structure of tight junctions described at the junction of two motile cells by Matsuda and co-workers [42]. We also noted the appearance of nuclear claudin-1, accompanied by a significant decrease in both cell number and the proportion of cells in G1, as well as an increase in nuclear and cytoplasmic volume that is a feature of the progression of cells through the cell cycle [43]. Significantly, neither the change in claudin-1 distribution, nor the decrease in cell number seen in cells transfected with *PHF11*-specific siRNA was dependent on prior treatment with poly(I:C).

Colon carcinoma cells show an increase in the cytoplasmic expression and nuclear localization of claudin-1 in primary tumours and metastases and knock-down of claudin-1 expression in cultured cell lines inhibits cell migration [44]. Several other membrane and tight-junction proteins also traffic between the membrane and the nucleus; the protein Zona Occludens 2 (ZO-2) interacts with several transcription factors and structural proteins in the nucleus to regulate cell growth and proliferation (for review see [45]). The exact role of claudin-1 in the nucleus is not known. We suggest that the change in cellular distribution of claudin-1 in HaCaT cells transfected with *PHF11*-specific siRNA might not be a direct effect of PHF11 knock-down, but is instead a result of differences in cell proliferation between cells transfected with control or *PHF11*-specific siRNA.

A genome-wide screen using RNA-interference gene knock-down was recently carried out in mice to identify genes involved in normal and oncogenic growth during skin development. This analysis identified *PHF11* as one of 1800 genes considered essential for normal growth [46]. It is interesting to note that an allele in the 3’ untranslated region of *PHF11* that is associated with asthma and dermatitis [5] reduces the expression of *PHF11* [1, 7] and that this is correlated with reduced binding of the transcription factor Oct-1 [7], which is highly expressed in epithelial cells. In experiments described here, we suggest that siRNA knock-down of *PHF11* expression led to a reduction in cell viability [2] and/or a slowing of cell proliferation that resulted in a sub-confluent monolayer at day 7 of culture, resulting in fewer cells arrested at G1 in the cell cycle at his time point.

In T-cells, PHF11 is a transcriptional co-activator of NF-κB, with knock-down of PHF11 associated with decreased binding of NF-κB to the *IFNG* promoter and decreased NF-κB-dependent transcription [1, 2]. Over-expression of PHF11 increased class-switch recombination to IgE in activated B-cells and this was correlated with increased binding of NF-κB [3]. Epidermal inflammation is regulated by NF-κB-dependent cross-talk between keratinocytes and infiltrating immune cells, while epidermal hyperplasia can be induced by dysregulation of NF-κB in keratinocytes alone [47]. It has been shown that NF-κB is constitutively active in HaCaT keratinocytes, resulting in increased apoptosis in response to ultraviolet light [48]. Despite the constitutive activation of NF-κB in HaCaTs, the sensitivity of this cell line to apoptotic stimuli is thought to reflect low NF-κB transcriptional activation [49]. As PHF11 potentiates NF-κB regulated transcription in lymphocytes [1, 2], the reason for the increase in *IL8* RNA associated with knock-down of PHF11 in HaCaT cells is less clear, given that *IL8* expression is increased in HaCaT keratinocytes through an NF-κB-dependent pathway [50]. Significantly, a microarray analysis of poly(I:C)-stimulated THP-1 monocytes also showed an increase in *IL8* RNA following *PHF11* knock-down (G. Jones, unpublished data), supporting the idea that PHF11 may be a negative regulator of poly(I:C)-induced *IL8* expression.

In this regard it is interesting to note that *PHF11* is adjacent to the gene *SETDB2* that encodes a histone methyltransferase that increases histone methylation on lysine 9 of histone 3 [51], a histone modification involved in gene repression. A co-transcript that may express a *PHF11/SETDB2* fusion protein has been reported in mouse [52] and human [53] cells. We are currently investigating whether epithelial cells express such a transcript and whether this transcript would include a functional histone methyltransferase domain.

## Conclusions

This report describes the regulation of *PHF11* by poly(I:C) in the HaCaT keratinocyte cell line and is consistent with other reports showing *PHF11* is an interferon stimulated gene that is induced by viral infection. Given that the knock-down of *PHF11* expression in HaCaT keratinocytes led to an increase in IL-8 expression immediately after poly(I:C) treatment whereas the change in cell number and the cellular distribution of claudin-1 occurred later and were independent of poly(I:C), *PHF11* may have several roles in the epithelial response to infection and injury.

## Abbreviations

IgE: immunoglobulin E
PHF11: plant homeodomain finger protein 11
TLR3: Toll-like receptor 3
IL-8: interleukin-8
poly(I:C): Polyinosinic:Polycytidylic Acid
